# Malpractice Claims and Ethical Issues in Prison Health Care Related to Consent and Confidentiality

**DOI:** 10.3390/healthcare10071290

**Published:** 2022-07-12

**Authors:** Oana-Maria Isailă, Sorin Hostiuc

**Affiliations:** 1 Department of Legal Medicine and Bioethics, Faculty of Dental Medicine, “Carol Davila” University of Medicine and Pharmacy, 020021 Bucharest, Romania; sorin.hostiuc@umfcd.ro; 2 “Mina Minovici” National Institute of Legal Medicine, 042122 Bucharest, Romania

**Keywords:** malpractice, prison, health care, consent, confidentiality

## Abstract

Respecting the consent and confidentiality of a patient is an underlying element in establishing the patient’s trust in the physician and, implicitly, obtaining the patient’s compliance. In particular, cases of inmate patients require increased attention in order to fulfill this goal against a background of institutional interferences, which, in certain situations, may endanger the autonomy of the physician and their respect for the inmate’s dignity. The purpose of this article is to depict the characteristics of consent and confidentiality in a prison environment, in special cases, such as hunger strikes, violent acts, HIV testing, COVID-19 measures, and drug use, bringing into focus the physician and the inmate in the context of the particular situation where the target is disciplining someone in order for them to conform to social and juridical norms. Respecting the dignity of the inmate patient requires an adequate approach of informed consent and confidentiality, depending on each case, considering the potential unspoken aspects of the inmate’s account, which can be key elements in obtaining their compliance and avoiding malpractice claims.

## 1. Introduction

Providing medical care is not always facile, and the outcome depends on the physician, the patient’s pathology, and the patient as a person [1]. The detention environment is an additional element that can have repercussions on the medical act. Medical errors can occur at the treatment planning stage or the treatment execution stage [2].

Consent and confidentiality are among the basic elements of medical practice, which can become problematic for inmate patients [3]. In accordance with General Assembly resolution 37/194 of the United Nations, ”Health personnel, particularly physicians, charged with the medical care of prisoners and detainees have a duty to provide them with the protection of their physical and mental health and treatment of disease of the same quality and standard as is afforded to those who are not imprisoned or detained” [4].

Situations in which healthcare personnel examine and treat inmates, according to the Council of Europe [5] and the WHO [6], are set out in Table 1. 

The percentage of inmates in need of terminal palliative care is much higher compared to the general population; an aspect which can be a direct consequence of the accelerating pathological processes and accentuating fragility of the patient in the context of measures of freedom deprivation [7]. 

Elements of informed consent which, in theory, are well-regulated both legally and morally can, in practice, present contextual discriminations given the freedom deprivation measures that have the potential to cause the patient to resort to aggression, dissimulation or simulation, lack of interest in the medical information, autolytic attempts, etc. [3]. Thus, informed consent can have questionable validity, with consequences on the medical act.

Therapeutic alliances in detention settings have questionable value due to the inherently coercive nature [8] which, given the potential mitigation of detention measures in certain diseases, can determine the patient’s tendency to exaggerate symptoms or even simulate the pathology [9].

The health issues of inmates can be present before incarceration or incurred during detention, an element which is also important in adequately approaching the inmate patient [6,10]. Furthermore, in-prison violence is a potentially significant factor causing additional morbidity or even mortality, either independent or in association with other disorders; thus, to ensure adequate consent and confidentiality, it is mandatory as a first measure to identify groups who are vulnerable to discrimination and victimization (ethnic groups, sexual and religious minorities, minors, inmates with mental health issues) [6].

The purpose of this unsystematized review is to analyze the particular aspects of consent and confidentiality in the case of the adult inmate patient and to suggest approaches that maximize the autonomy as well as the confidentiality of the inmate patient in their best medical interest.

## 2. Malpractice Claims in Prison Health Care

Claims of malpractice involve a breach of a professional obligation on the part of the physician causing healthcare-related damage. They might be caused by suboptimal medical management of the disease, errors in obtaining informed consent, deliberate indifference, etc. [11]. Informed consent and the confidentiality of medical data are essential to obtain the patient’s trust and compliance, both for proper diagnosis and treatment. Malpractice, referred to by some authors as medical negligence, may be commissive (acts or wrongdoing), omissive (not performing medical interventions that should have been performed), caused by faults in selecting the best alternative, or failures in supervising or exercising due diligence [12]. According to Vaughn and Collins, the underlying medical malpractice in prisons was related to medication, medical procedures, diagnosis, or undertreatment of a serious medical condition [13]. According to Tripathi et al., concerning allegations of malpractice of detainees with dermatological pathologies, one of the reasons for the accusation was the lack of informed consent [14]. Jeng et al., following the analysis of the allegations of malpractice of detainees who required ophthalmic treatments, observed that most of the reasons given referred to inadequate or incomplete treatment or refusal of treatment [15]. Such charges may also be subject to situations of non-confidentiality or interference between non-compliance and confidentiality or inadequate informed consent. In a recent analysis of allegations of malpractice in orthopedics, Lv et al. identified inadequate monitoring of patients, inadequate performance of procedures, and the inability to communicate with the patient, as main causes leading to the transmission of unclear medical information [16].

## 3. Consent and Confidentiality in Prison Health Care

### 3.1. Informed Consent

Informed consent implies transmitting information of interest to the patient so that they understand and are able to take well-thought decisions on their medical care. The physician has the responsibility to help the patient understand their medical state and their treatment options. The process of obtaining informed consent is based on adequate communication between the physician and the patient. As a result, the patient consents to undergo a specific medical procedure in the case where the patient has the capacity to consent and wishes to partake in taking of medical decisions, otherwise, surrogate informed consent is obtained.

This requires evaluating the patient’s capacity to understand the relevant medical information, their voluntary capacity, and their decision-making capacity. The physician informs the patient of aspects referring to the diagnosis and therapeutical options, including their nature, purpose, associated risks, costs, availability and the evolution of the disease in the absence of treatment or under the condition of renouncing treatment. 

Only in situations that are medical emergencies and the patient does not have the capacity to partake in the decision-making process and surrogate informed consent cannot be obtained, due to the nature of the patient’s condition, urgent medical treatment can be initiated without prior informed consent; however, informed consent will be obtained by the physician as soon as possible in order to continue providing medical care [3,17]. 

The general recommendations of the Council of Europe regarding informed consent stipulate: “Unless inmates suffer from any illness which renders them incapable of understanding the nature of their condition, they should always be entitled to give the doctor their informed consent before any physical examination of their person or their body products can be undertaken, except in cases provided for by law. The reasons for each examination should be clearly explained to and understood by the inmates. The indication for any medication should be explained to the inmates, together with any possible side effects likely to be experienced by them. Informed consent should be obtained in the case of mentally ill patients as well as in situations when medical duties and security requirements may not coincide, for example, refusal of treatment or refusal of food. Any derogation from the principle of freedom of consent should be based upon the law and be guided by the same principles which apply to the population as a whole”.

In order to comply with these goals, medical staff need to properly understand the characteristics of the patient, taking into account the aspect of detention. The overlapping of inmate and patient implies some aspects referring to the person’s psycho-social background, such as social marginalization, a very low socioeconomic level, a low level of schooling, reduced intellectual efficiency, aggression, impulsivity, reduced tolerance to frustration, personality disorders, and disorders resulting from substance use [3,18]. All these characteristics, particularly in the context of custodial measures, emphasize the patient’s need to have their autonomy respected within the patient–physician relationship to obtain their compliance and their trust in the physician. Informed consent is the basis of the medical act centered on the patient’s autonomy, with all its valences: understanding, volunteerism, and decision making capacity [19]. 

Usually, informed consent is obtained in writing, but in the case of illiterate patients, this is not possible. Moreover, even if the patient can read, information comprehension tests are required. A limited comprehension capacity is not necessarily reflected in the patient’s ability to read. In inmates, mental illness, substance use, and traumatic brain injuries with temporary or permanent repercussions on the person’s cognitive functions were also found, as well as low level of schooling and literacy [3,20]. Detention in itself, for example in the context of prolonged isolation, can determine deficiencies in their capacity to comprehend [21].

The traits of a patient which can raise suspicion about insufficient understanding of the transmitted medical information are presented in Table 2.

To facilitate the comprehension of the medical information transmitted, various methods can be used, such as multimedia presentations, simplifying the informed consent form, excluding redundant information, using concise phrases, etc. [3].

**Figure 1:**
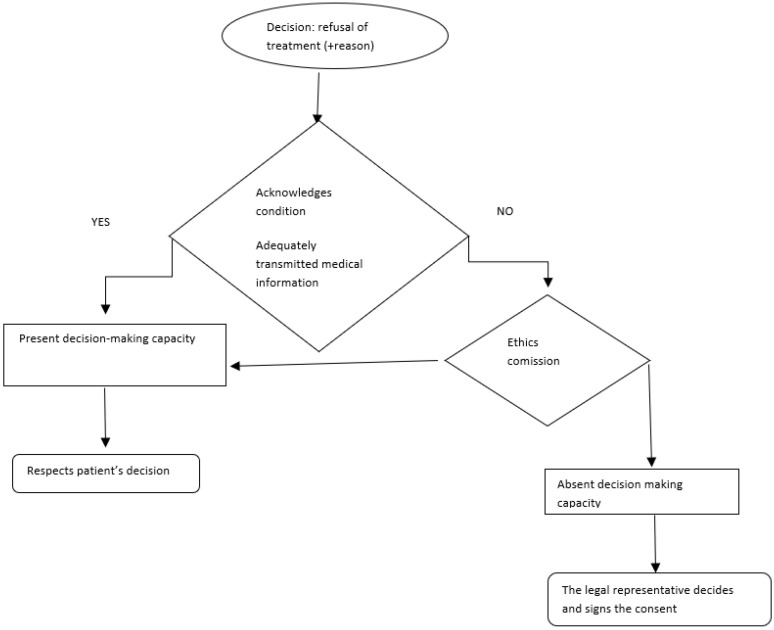
The algorithm in case of refusal of medical treatment according to Appelbaum [24].

In the case of refusal of treatment, according to Appelbaum [24], approaching the situation can be depicted as shown in Figure 1.

Furthermore, once the transmitted medical information is understood, the decision of the inmate patient must be voluntary, without the existance of any external constraint. For example, if a patient with acute appendicitis refuses surgical intervention as a measure of protest to their received sentence and the psychiatric evaluation indicates they have the capacity to make decisions, the patient understands the pathology and the consequences of the refusal of their health. Thus, the physician must respect the patient’s decision, even if it is to their detriment, and must assist them further and to make sure that the patient understands they can request surgical treatment to their benefit at any time [23].

Managing the patient’s refusal entails an adequate approach to factors such as the accuracy of the information, cultural or religious convictions of the patient, the physician–patient relationship model (adopting a slightly more paternalistic attitude, augmenting an informative type of relationship through two-way exchanges of information and experience between doctor and patient), and adopting an empathetic, benevolent attitude, interested in inter-relationship with the patient. In the event of the patient’s refusal of the indicated treatment, the physician must not take on a coercive attitude, which would undermine the patient’s autonomy, and must not ask for the patient’s legal representatives to sign the consent or for the ethics commission to intervene in situations when the patient has the capacity to make decisions.

In any situation in which a patient capable to make decisions refuses an intervention with the purpose of diagnosis or treatment, the physician is required to document this by completing an informed refusal form in the presence of a witness who must countersign. In this informed refusal form, the patient must acknowledge the risks and benefits of the treatment, this being an additional measure through which the physician ensures that the patient has understood the information [25].

### 3.2. Confidentiality

Confidentiality is the main element in respecting the patient’s privacy and, at the same time, it helps consolidate the patient’s trust in the physician and the healthcare system. The physician, except for particular contexts required by law, has the obligation to maintain the confidentiality of the patient’s medical history, diagnosis, and treatment. The situations where confidentiality is to be excepted must be clearly defined and justifiable by the legal framework and medical ethics, and brought to the patient’s attention [26,27]. Generally, the patient has the right to decide in regard to disclosing confidential information. Taking this into account, privacy and, implicitly, confidentiality are not absolute values and can be limited by more important moral considerations [28]. Situations that bring into discussion the disclosing of confidential information by the attending physician without the consent of the patient being required, besides the situations regulated in the interest of justice (such as making the patient’s medical history files available to the courts upon request), refer mainly to the significant danger posed by the patient towards themself and/or a third party. The reasoning behind disclosing confidential information must include a clear definition of the problem, collecting the relevant information, identifying options of action, and comparatively evaluating them, the decision, and the evaluation of the consequences [29].

According to HIPAA (Health Insurance Portability and Accountability), legal limits of confidentiality [29] can be classified as presented in Table 3.

Against the background of custodial measures, the stages of this reasoning become more complex, especially considering that the prison physician is also subordinated to the prison management, and situations may occur in which the prison administration requests confidential information on the prisoner. In this context as well, the physician must emphasize the physician–patient relationship and act in a way that is in the best interest of the patient [6,30]. According to WHO, “The results of medical examinations and tests undertaken in prison with the patient’s consent as part of clinical care must be treated with the same respect for confidentiality as is normal according to medical ethics in general medical practice”; “Prison physicians should avoid dual roles with the same patient. To avoid as far as possible any confusion about the role of the doctor in medical examinations and treatment in the caregiving role and in other functions (such as providing medical expertise, and forensic records), the doctor should make it clear to the patient at the outset of the consultation that medical confidentiality will not apply to the results of any medical examinations and tests undertaken for forensic purposes” [6]. Practicing medicine in a prison environment involves multiple loyalties. The duty to ensure the best care of the patient and promote health may be at odds with the priorities imposed by the correctional facility’s management. For example, the physician may be asked to disclose the HIV status of a patient [6,31].

Activities that would contravene the ethical principles involving the attending physician/medical staff from a correctional facility in a relationship with the inmate, without the purpose of evaluating, protecting, or improving their state of health [4], which also includes the disclosure of confidential information, may include those presented in Table 4. 

The opening of confidential information acquired during the exercise of the profession (the professional secrecy) can have consequences such as: breaking the therapeutic alliance, precipitation of aggressive phenomena, stigmatization of the patient by society, litigation of the doctor by violating professional secrecy, and loss of the right to free practice [33].

Dual-loyalty is an important dilemma in medical practice. In order to solve such dilemmas in correctional environments, it is recommended that medical professionals who are not responsible for the care of detainees (e.g., forensic pathologists) undertake the tasks imposed by the courts or the security system. Physicians who practice in prisons have a moral duty to care for detainees which must prevail over other institutional or interinstitutional interests [32]. However, in some situations, precisely through the lens of the duty of medical care for the detainees, they must resort to dual-loyalty. Such contexts refer mainly to infectious–contagious diseases when the pathology of the patient detainee endangers the health of third parties. Furthermore, the patient’s autolytic ideation places the doctor in a scenario where the solution to protect his life and health involves reporting the case after a prior assessment of suicide risk. The case report aims to remedy the elements underlying the autolytic ideation (e.g., internal changes in prison arrangements). This scenario, in the conditions in which a patient with adequate decisional capacity would not agree with the reporting of the situation, brings into question the paternalistic breach of confidentiality, promoting the good of the patient over autonomy [34]. A similar approach is required if the patient states their intention to kill/assault, and, following prior assessment of the risk of danger, it is necessary to protect the health of third parties. The prioritization of the good of the third person prevails with the analysis of the situation in case that no maleficence is generated to the patient. The opening of professional secrecy may take place in the certain scenarios (Table 5).

**Figure 2:**
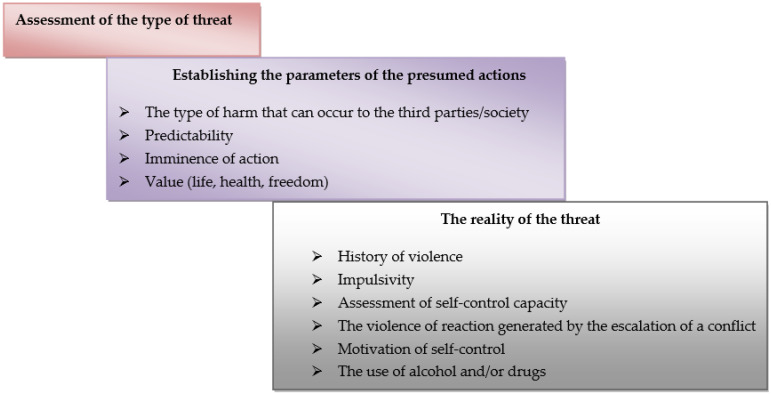
The algorithm if the patient states they intend to harm third parties [33,35].

If the patient poses a threat to third parties, the following assessment algorithm is recommended (Figure 2).

Violation of confidentiality in unnecessary situations without prior explanation, may in the future, lead the patient to conceal medical data essential for a proper therapeutic approach.

## 4. Particular Scenarios Physician–Inmate Patient

### 4.1. Hunger Strike

The inmate’s refusal to eat can have multiple causes, for example, religious beliefs, different somatic or psychiatric pathologies (which can be resolved by treating the main illness), or as a sign of protest. The hunger strike is a common type of protest which is based on the person’s need to obtain social resonance. In prison environments, this situation is more complex and generates difficulties for the correctional staff administration as well as for the attending medical staff. The inmate resorts to a hunger strike usually because of a desire to change a certain juridical or administrative situation they consider unjust or damaging to their interest [36]. From a deontological standpoint, the will of a person who has the capacity to make decisions and does not endanger third parties must be respected. According to the WHO, the conflict of autonomy and beneficence will be approached according to the provisions of the World Medical Association within the Declaration of Malta (WHO refers to the 2006 version of the declaration), which states for cases of hunger strikes in custody: respecting the autonomy of the patient after a prior examination of their decision-making capabilities, ensuring that the inmate has fully understood the medical consequences of this method of protest, continuing to ensure necessary medical treatment (for example, treating pain and infection), examining the inmate daily and administering liquids, vitamins, glucose and nutrients if the inmate consents, to avoid irreversible, even lethal consequences. Force-feeding a person is not acceptable in any given situation, and is considered degrading and inhumane. Artificially feeding with explicit consent or because of the inmate’s implicit necessity is acceptable from an ethical point of view. In addition, in this type of situation, confidentiality must be maintained but can be disclosed if the person wishes, or in order to prevent serious harm. The attending physician’s duty is to make successive and objective reports on medical criteria, through which they inform the judicial authorities of the evolution of the inmate’s state of health with the prospect of taking adequate decisions towards the welfare of the person in danger while being well informed [6]. The updated 2017 version of the Declaration of Malta, in concurrence with prior provisions, mentions in regard to confidentiality that, if the patient does not wish to disclose confidentiality, the attending physician must inform the patient of the potential situation which they will impose this against the patient’s wishes [28]. If a medical examination is not consented to, the physician must respect this. In severe cases, taking into account the particular context of prison measures, the fact that their wishes may have been written while pressured, or the fact that their wishes could radically change once losing mental competency, the physician must act in the best medical interest of the patient, taking on a paternalistic approach [37].

Furthermore, according to the WMA-Declaration of Malta 2017 “Physicians may rarely and exceptionally consider it justifiable to go against advance instructions refusing treatment because, for example, the refusal is thought to have been made under duress. If, after resuscitation and having regained their mental faculties, hunger strikers continue to reiterate their intention to fast, that decision should be respected. It is ethical to allow a determined hunger striker to die with dignity rather than submit that person to repeated interventions against his or her will. Physicians acting against an advanced refusal of treatment must be prepared to justify that action to relevant authorities including professional regulators” [37]. However, this approach is still met with controversy by some authors and it brings to the forefront the duty of the state to protect the lives of the inmates [38] as stated by the provisions of the European Court of Human Rights “a measure which is a therapeutic necessity from the point of view of established principles of medicine cannot in principle be regarded as inhuman and degrading. The same can be said of force-feeding which is aimed at saving the life of a particular detainee who consciously refuses to take food. The Court must nevertheless satisfy itself that the medical necessity has been convincingly shown to exist. Furthermore, the Court must ascertain that the procedural guarantees for the decision to force-feed are complied with. Moreover, the manner in which the applicant is subjected to force-feeding during the hunger strike must not trespass the threshold of the minimum level of severity envisaged by the Court’s case law” [39].

Given the above, in the patient approach algorithm, respecting the patient’s autonomy is paramount through consent and confidentiality, with medical supervision of the inmate and their adequate information, including concerning provisions of national law, which at a certain time may require a paternalistic approach [31].

### 4.2. Consecutive to Acts of Violence

Violence in prisons, with all of its possible forms, often remains unreported for fear of possible retaliation [6]. Sexual violence, in particular, is much more difficult to quantify because of the stigma the victim faces in the prison environment and the possible increase in the abuse [40].

Moreover, it is a way in which STDs are transmitted, since, for example, HIV infection rates are higher than in the general population. Furthermore, the victim can have depression, PTSD, unhealthy behaviors, and autholityc ideation [40,41]. A helping role in reporting and implicitly preventing violence in prison environments is accessibility to medical care, which, according to a study conducted by Ross et al., also creates a more positive atmosphere [42].

The physician, when treating such a patient who has traumatic lesions or clinical manifestations which can stem from possible abuse, must record these aspects in the medical chart, including the patient’s statements (when present), and disclose confidentiality by reporting to the supervising authorities in order for them to take measures in this regard, while also informing the patient. These steps are required since, once the aggressor discovers that the abuse has been reported, there is a higher risk of retaliation/more abuse on the victim. Furthermore, medical staff must have a framework to report cases of violence to neutral state organizations as well as outside of the prison environment [6]. However, within “prison culture”, cases of violence, especially cases of sexual violence, are rarely reported to medical staff [43]. 

### 4.3. HIV Infection

International provisions for these types of situations highlight the importance of the person’s consent regarding testing and treating, as well as in regard to disclosing confidentiality. Despite the practice within some penitentiary units which claim the prevention of transmission through compulsory testing [35], compulsory testing is rejected. The efficiency of testing programs was demonstrated only in cases where there were also adequate therapeutic and counseling resources present, which ensured the patients’ compliance [44]. 

### 4.4. Other Contagious Diseases

In the case of contagious diseases, additional measures are required to protect detainees from contamination. This involves the detection of contaminated persons, their treatment, and the application of prophylactic measures for people who have not contracted the pathology. Measures taken in such situations are legally enforceable at each national level. For example, related to the COVID-19 pandemic context, the study by Vella et al. in Italy found a lower number of COVID- positive cases among detainees than among the general population, which reflected the effectiveness of the measures taken, including vaccination [45]. In the same sense, Pagano et al. found that measures such as screening and safe isolation of COVID-positive or COVID-suspect detainees prevented the spread of the virus [46]. These issues again call into question the peculiarities of consent and confidentiality, given the danger to the health of third parties with whom they may come into contact and who require protective measures. The physician has the duty to report the medical condition with the prior information of the patient, without abandoning him, respecting the principle of dual-loyalty [47]. Regading the issue of vaccination, according to the WHO “Vaccination mandates can be ethically justified; however, their ethical justification is contingent upon a number of conditions and considerations, including the contexts within which they are implemented” [48]. In detention environments, given the interpersonal proximity, mandatory testing, case reporting, and mandatory vaccination of people who do not have contraindications, it can be justified to maximize group and individual benefits, according to the utilitarian principle. However, the autonomy of the person should not be omitted, as prior information campaigns are required [49]. Regarding the individual well-being of the patient, COVID-19 infection can endanger even the life of the detainee, who, through the prism of detention, can present other pathologies, for example, tuberculosis, with a higher incidence in such environments [50]. Unfavorable living conditions prior to detention and unhealthy behaviors such as drug use, more common in detainees, increase the risk of developing pathologies such as TB, HIV, HCV [51].

### 4.5. Drug Use

Drug use is a priority issue for public health as well as for prison environments. However, statistics referring to this aspect are limited because it is a delicate subject correlated with eventual breaches in the security of detention and with subsequent punitive measures for the inmates. Some prisons have mandatory testing programs which are periodically randomized [52,53]. In the case of patients under the influence of drugs, obtaining valid informed consent becomes questionable because they can have reduced or absent decision-making capacity. They can simulate understanding the transmitted medical information or can refuse the proposed treatment without a coherent reason, which can require starting the procedure to obtain informed consent from the person’s legal representative [54]. Furthermore, in the absence of adequate information in regards to the purpose of collecting biological evidence for a pathology without a toxicology background, they can unjustifiably refuse because they are afraid of subsequent toxicology exams. In the situation where the medical welfare of the patient requires testing for drugs, aspects regarding confidentiality and consent will be approached according to the existing provisions in the local law [35].

## 5. Conclusions

Absence of physical freedom should not interfere with the freedom to decide on one’s own health. Detention should not be perceived as an eradication of a person’s autonomy, their own will, and their freedom of thought. The attention of world organizations in this regard has led to provisions that differ in some places but that bring to the fore the dignity of the human being. The physician, through the prism of the profession, is the one who should watch over these desideratum. Beyond the international recommendations, each national legal framework prevails, based on which the medical conduct in case of accusation of malpractice is evaluated.

In prison, the inmates’ state of health requires constant attention. In this environment, with all the contextual and juridical peculiarities, the physician has the duty to act in the patient’s best interest. Respecting the dignity of the inmate patient requires an adequate approach of informed consent and confidentiality, depending on each case and on the legal framework, considering the potential unspoken aspects of the inmate’s account, which can be key elements in obtaining their compliance and avoiding malpractice claims. 

## Figures and Tables

**Table 1:** Situations in which healthcare staff examine/treat inmates according to the Council of Europe [5] and the WHO [6].

Checking Nutrition Status and Hygiene	
Following acts of violence	Suicide, attempted suicide, self-harm Physical altercations between inmates Psychological violence: threats, bullying, humiliation Sexual assault among inmates or sexual assault committed by correctional officers or other prison staff Torture or ill-treatment applied to inmates by correctional officers or other prison staff Isolated acts of violence or general acts of violence (riots) of inmates on prison staff
Communicable diseases	HIV, hepatitis, tuberculosis Influenza, measles, mumps, rubella, diphtheria Sexually-transmitted infections Ectoparasites
Noncommunicable diseases	Cardiovascular diseases, cancer, diabetes, chronic respiratory diseases
Unhealthy behavior/risk factors	Smoking, alcohol use, drug use, inadequate physical activity, inadequate diet
Mental health problems	Anxiety disorder, depression, phobia, eating disorders
Oral health problems	Dental stomatitis, dental decay, dental erosions, maxillo-facial fractures

**Table 2:** Traits that can affect the patient’s capacity to understand the medical information transmitted, according to British Medical Association and Law Society [22] and [23].

Aspect and behaviors	Agitated patient Mood disorders Cognitive disorders
Speech	Silent patient—can suggest depression Tangential, high-speed speech—can suggest hypomania
Mood	Depression and hypomania—distort the perception of the future Emotional instability—patients who have difficulty choosing a certain treatment
Thoughts and perception	Perception disorders (illusions, hallucinations) or overstated ideas lead to alteration of decision-making capacity
Cognition	Attention and concentration disorders Distraction
Memory	Cognitive disorders, memory disorders
Intelligence	Reduced intellectual abilities due to lack of education
Orientation in space and time	Cognitive disorders, disorders from substance use
Insight	Prior understanding of the presented medical issue

**Table 3:** Legal limits of confidentiality, according to HIPAA [29].

Duties to warn third parties of harm	Measures to warn a third party, depending on the level of the risk posed by the patient (and implicitly the risk of harm), in regard the danger represented by the patient
Duties to report various medical conditions	Infections with agents etiologically specific to bioterrorism (eg. anthrax, smallpox, plague, botulism, tularemia, viral hemorrhagic fevers) New epidemic diseases (eg. SARS-CoV 2 infection), the person’s capacity to operate vehicles Patients who are victims of domestic abuse (mainly minors and elderly persons)
Duties to inform legal guardians and other surrogates about the care of minors and other incompetent patients	Not applicable in the case of emancipated minors

**Table 4:** Activities that can be asked of the attending physician and which contravene ethical principles, placing the inmate at a medical disadvantage [4,6,32].

Criminalistic evaluations
Disclosing the patient’s medical data to other persons without their consent
Assisting bodily searches
Assisting the collection of biological evidence (blood and urine) for security reasons
Supplying medical expertise measures to apply disciplinary measures Assisting/participating in physical constraint in the absence of medical criteria to warrant it
Assisting/participating in physical or capital punishment
Torture
Forced feeding

**Table 5:** Scenarios that require the opening of professional secrecy [33].

With the patient’s consent	Some information that may be used to the detriment of the patient must be considered and evaluated, an aspect that they may not understand or be aware of
Implicit	In dealing with other workers who need to provide the patient with adequate living conditions to protect their health (for example: the cook must know that the prisoner is allergic to a certain type of food)
Even if the patient did not agree	Strictly on the basis of the legislative framework
Without informing the patient	When the patient endangers the physician or a third party
